# Noninvasive Monitoring of Dynamical Processes in Bruised Human Skin Using Diffuse Reflectance Spectroscopy and Pulsed Photothermal Radiometry

**DOI:** 10.3390/s21010302

**Published:** 2021-01-05

**Authors:** Ana Marin, Nina Verdel, Matija Milanič, Boris Majaron

**Affiliations:** 1 Faculty of Mathematics and Physics, University of Ljubljana, 1000 Ljubljana, Slovenia; ana.marin@fmf.uni-lj.si (A.M.); matija.milanic@fmf.uni-lj.si (M.M.); 2 Department of Complex Matter, Jožef Stefan Institute, 1000 Ljubljana, Slovenia; nina.verdel@ijs.si

**Keywords:** traumatic bruise, contusion, bilirubin, diffuse reflectance spectroscopy, integrating sphere, pulsed photothermal radiometry, inverse Monte Carlo

## Abstract

We have augmented a recently introduced method for noninvasive analysis of skin structure and composition and applied it to monitoring of dynamical processes in traumatic bruises. The approach combines diffuse reflectance spectroscopy in visible spectral range and pulsed photothermal radiometry. Data from both techniques are analyzed simultaneously using a numerical model of light and heat transport in a four-layer model of human skin. Compared to the earlier presented approach, the newly introduced elements include two additional chromophores (*β*-carotene and bilirubin), individually adjusted thickness of the papillary dermal layer, and analysis of the bruised site using baseline values assessed from intact skin in its vicinity. Analyses of traumatic bruises in three volunteers over a period of 16 days clearly indicate a gradual, yet substantial increase of the dermal blood content and reduction of its oxygenation level in the first days after injury. This is followed by the emergence of bilirubin and relaxation of all model parameters towards the values characteristic for healthy skin approximately two weeks after the injury. The assessed parameter values and time dependences are consistent with existing literature. Thus, the presented methodology offers a viable approach for objective characterization of the bruise healing process.

## 1. Introduction

Assessment of the bruise age is a frequent requirement in investigations of offences involving physical abuse. The most common approach is based on temporal changes in skin discoloration due to dynamic processes involving extravasated hemoglobin and products of its biochemical decomposition. Consequently, bruises initially appear as red or pink discoloration which transforms relatively quickly into bluish/purple, followed by grey/brown, green, and yellow hues, until the resolution 2–3 weeks post injury [1]. However, the current protocol relies exclusively on visual inspection and subjective assessment by a forensics expert. This results in limited accuracy of the assessed age, due to uncontrolled variability of lighting conditions and individual skin characteristics related to the subjects’ age, gender, race, sun tanning, anatomical location, etc. [1,2,3].

Aiming at development of a more accurate and robust method for bruise ageing, various optical techniques were proposed and tested by monitoring temporal evolution of self-healing bruises, including colorimetry [4], bilirubinometry [5], diffuse reflectance spectroscopy (DRS) [6,7,8], digital photography [9,10], and hyperspectral imaging in visible spectral range [11,12].

DRS is a popular technique in biomedical research, offering a useful insight into the presence of various skin chromophores, including various hemoglobin species and their derivative, bilirubin [13]. However, the DRS spectra represent a lumped sum of the light scattered and absorbed at various subsurface depths. Quantitative assessment of the chromophore contents in a multi-layered organ, such as skin, thus requires additional information about their depth distribution [14,15]. This could be gained, e.g., by performing spatially or temporally resolved DRS, or assessing the thicknesses of the characteristic skin layers by independent measurements (using optical coherence tomography, confocal microscopy, etc.).

Pulsed photothermal radiometry (PPTR) might represent another viable approach to obtain the required information on depth distribution of the relevant skin chromophores. PPTR involves measurements of transient changes in mid-infrared emission from the sample surface after exposure to a short light pulse. Analysis of such radiometric signals allows assessment of the irradiation-induced temperature depth profile, thus revealing the depths of absorbing structures inside an optically scattering medium [16], including blood-related pathologies in human skin in vivo [17,18].

However, PPTR alone cannot provide a unique assessment of the contents of multiple chromophores present in human skin [14,18]. Therefore, our group has recently introduced a combined application of DRS and PPTR, which enables simultaneous assessment of the structure and composition of human skin in vivo [19]. This is achieved by simultaneous fitting of the DRS spectra and PPTR signals with predictions from a dedicated numerical model of light-tissue interaction (i.e., inverse Monte Carlo approach). The approach was tested by comparing the assessed properties of healthy skin with literature data, and by co-registration with multi-photon microscopy [19,20]. In addition, it was successful in revealing the changes in human skin induced by temporary obstruction of peripheral blood circulation, acute and seasonal sun tanning [19], and most recently also laser treatment of unwanted tattoos [21].

In the present study, we investigate whether the same approach could be adapted for objective monitoring of dynamical processes in self-healing traumatic bruises. To that end, one essential augmentation involves accounting for two additional chromophores, i.e., carotenoids in intact and bilirubin in bruised skin [22]. Second, the thickness of the papillary dermal layer is adjusted individually for greater flexibility of the skin model; and third, in order to improve the robustness of our analysis, an intact skin site in the vicinity of the bruise is characterized first by the four-layer model of healthy skin [23]. Only selected parameters of the optical model are then allowed to vary to match the data obtained from the bruised site. This step is based on the assumption that the observed changes of the bruise color result primarily from mass diffusion of extravasated hemoglobin and its subsequent biochemical decomposition and removal. Meanwhile, the gross structure and composition of the skin, especially the epidermis, should remain relatively intact.

As we show further below, the described analysis reveals clearly and robustly the substantial increase of the dermal hemoglobin content and drop of its oxygenation level compared to the healthy baseline values in all tested bruises. This is followed soon by the emergence of bilirubin, which is not present in healthy skin. Two or more weeks after the injury, all variables relax towards the values characteristic for intact skin. The indicated time dependences, albeit obtained from independent analyses of the data collected at different time points, are relatively smooth and mutually consistent. They are also in good agreement with the trends and values from independent reports by other research groups, as well as general understanding of the underlying processes.

Systematic studies of the bruise healing dynamics, including their inter- and intra-subject variabilities, will be essential for development of future methodologies for robust and accurate assessment of the bruise age. We believe that the described technique presents one viable approach and may contribute to this process.

## 2. Materials and Methods

### 2.1. Human Volunteers

This report involves 3 volunteers (29–30 years old) with fair skin (Fitzpatrick types I–II). All presented minor or moderate bruises with an area of at least 1 cm^2^ and a known time of injury. All bruises occurred incidentally, as a result of sports activities or minor accidents (see Table 1).

### 2.2. Experimental Procedures

**Figure 1:**
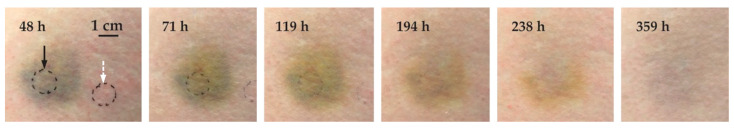
Photographs of the bruise in subject *a* at different times after the injury (see the labels), displaying the characteristic sequence of color changes. Dashed circles indicate the test sites on the bruised area and intact skin in its vicinity (black and white arrow, respectively).

The measurements were performed in 7 sessions at times between 13 and 376 h after the injury. Digital photographs of the bruised area were taken before each measurement to enable subsequent correlation of our results with the visual impression of discoloration. One example is presented in Figure 1.

Selected test sites on and near each bruise were gently shaved as necessary. The most superficial layer of dry epithelial cells was removed by tape stripping (10–15 repetitions) [24] to help ensure unimpaired heat diffusion to the skin surface as required for the PPTR measurements. The site was cleaned using medical-grade ethanol, rehydrated with physiological solution and left to dry.

Diffuse reflectance spectra in visible spectral range (400–700 nm) were acquired from the bruised and a nearby intact test site (see Figure 1) using an integrating sphere with internal light source and a sample opening with the diameter of 10.3 mm (ISP-REF by Ocean Optics, Dunedin, FL, USA). The spectral response of the spectrometer (USB4000, Ocean Optics) was calibrated using a white standard (Spectralon© by Labsphere, North Sutton, NH, USA). The signal-to-noise ratio (SNR) was improved by averaging of 100 spectra acquired at an integration time of 22 ms. The single-beam substitution error, which can be significant when using such small integrating spheres, was removed by numerical preprocessing of raw DRS data [25].

Next, the bruised and intact test sites were irradiated separately with a 1 ms pulse emitted from a medical-grade KTP laser (DualisVP by Fotona, Ljubljana, Slovenia). The emission wavelength of 532 nm is absorbed well in both epidermal melanin and dermal hemoglobin, and was found very suitable in our earlier studies involving either PPTR profiling or model-based characterization of human skin in vivo [18,19,20,26]. At the laser beam incidence angle of 15–20° with respect to the normal and effective spot size around 9 mm, the central radiant exposure was 0.20–0.25 J/cm^2^. Such radiant exposure levels are at the limit of Class 1 irradiation [27] and induce peak temperature rises below 2 K in fair human skin [26].

The subsequent mid-IR emission (wavelength range 3.5–5.1 μm) from the tissue surface was recorded by a fast IR camera (FLIR SC7500) for 5 s at a rate of 1000 frames per second. The transient radiometric signals were obtained from the recorded sequences by lateral averaging over a homogeneous area of interest (up to 8 mm^2^ in size) and subtracting the constant pre-pulse value. The manufacturer-provided calibration system was used for nonlinear conversion of the signal amplitudes to radiometric temperature values. Finally, 3–5 such signals obtained from the same test site were averaged to improve the SNR and reduce potential artifacts due to uncontrolled physiological processes, such as heartbeat or thermoregulation.

### 2.3. Optical Model of Intact Skin

We employ a four-layer optical model of human skin, consisting of epidermis, papillary and reticular dermis, and subcutaneous tissue, as developed and tested earlier in our group [19,22,23]. Thicknesses of the first three layers (*d*_epi_, *d*_pap_, and *d*_ret_) are fitted individually, while the subcutaneous layer is modeled as semi-infinite.

In shortest terms, the absorption coefficients of epidermis and dermis are computed by following the widely accepted formulae from Jacques [28]. They combine a fixed baseline spectrum and contributions due to layer-specific absorbers, i.e., melanin in the epidermis (with volume fraction *m*) and blood in both dermal layers (*b*_pap_ and *b*_ret_, respectively). The absorption coefficient of blood is calculated as a linear combination of the values for oxygenated and deoxygenated blood [29], with independent saturation levels for the papillary and reticular dermis (*S*_pap_, *S*_ret_). A small amount of blood is also allowed in the epidermal layer to account for the undulation of the epidermal-dermal junction (*b*_epi_).

In contrast with the earlier version of this model, the spectral interval of 475–525 nm is of particular relevance for this study because it contains significant absorption of the hemoglobin decomposition product, bilirubin. While bilirubin should not be present in skin of healthy adults, the same spectral range harbors also absorption of carotenoids, especially *β*-carotene [30]. Therefore, in order to get a meaningful assessment of the intact (control) site we include *β*-carotene (with concentration *c*_β_) in the dermal absorption coefficient, using the absorption spectrum as measured when diluted in lipids [13].

The absorption spectrum of the subcutaneous adipose tissue is adopted from Simpson et al. [31]. Although *β*-carotene can often be found in this layer, we do not account for it explicitly. The amount of incident blue/green light that interacts with this layer and is subsequently reemitted from the skin surface is, namely, negligible.

Scattering properties of the epidermis and dermis are optimized separately, according to the customary ansatz:

(1) $$\mu_{s}^{\prime }\left(\lambda \right)=a\;{\left(\frac{\lambda }{500\;nm}\right)}^{-p}.$$

Doing so was, namely, shown earlier to help improve the match between the model predictions and experimental data [19], in line with the common belief that scattering properties of skin can exhibit significant inter- and intra-personal variations [28]. The scattering properties of subcutaneous tissue are also adjusted, by varying a single amplitude parameter (*A*) [15].

The refractive indices are set to *n* = 1.45 for the epidermis, 1.37 for the dermis, and 1.34 for subcutis [32]. The epidermal and dermal scattering anisotropy factor is approximated as *g* = 0.62 + 29 × 10^−5^ λ/nm, while for subcutis it is set to 0.77 [32].

### 2.4. Optical Model of the Bruise

Traumatic bruises occur as a result of a blunt force impact, which ruptures blood vessels in lower dermis and/or subcutis. Hemoglobin, which is released from the extravasated red blood cells (RBC), then diffuses through the dense extracellular matrix. At the same time, it is gradually removed through the lymphatic system and biochemically transformed into biliverdin, which is in turn transformed rapidly into bilirubin [33]. In line with this paradigm, the model parameters that are allowed to change with respect to the baseline values (assessed from the preceding analysis of the nearby intact site) include the dermal blood contents and oxygenation levels (*b*_pap_, *b*_ret_, *S*_pap_, and *S*_ret_), now accounting for both vascular and extravasated hemoglobin.

In contrast with the version used for intact skin (Section 2.3), this model allows for presence of bilirubin (with concentration *c*_bili_) with the absorption spectrum as measured when diluted in human serum albumin [13]. In addition, we allow for variation of the dermal scattering amplitude, *a*_der_. This decision was based on numerous preliminary analyses [34] and realization that several dynamical processes involved in the bruise healing process may affect the scattering properties of skin, especially the dermis (see Section 4).

Note that, while the hemoglobin released at the hemorrhage site can diffuse in all directions, both DRS and PPTR are single-point measurements. Consequently, we can apply only one-dimensional optical models of skin with lateral uniform layers. For this reason we limit our measurements to the center of the visually homogeneous discoloration patch, where lateral gradients and mass diffusion should be minimal. Thus, our analysis approach is sensitive only to depth distribution of optical absorption and scattering. This is an important difference with respect to the wide-field imaging techniques, such as digital photography or hyper-spectral imaging, which provide laterally resolved information but often lack intrinsic sensitivity to the depth distribution of the relevant chromophores.

### 2.5. Numerical Modeling of Light-Tissue Interaction

Light transport and energy deposition of skin during our measurements are simulated using the weighted-photon multi-layer Monte Carlo technique (MCML) [35] in the massively parallelized implementation (using the GPU) as derived by Alerstam et al. [36]. Each simulation run involves launching of 5 × 10^6^ “photons” for each considered wavelength. Diffuse reflectance values are computed by accounting for the finite sample opening of the integrating sphere used in our measurements [15].

By considering the thermal properties of the involved skin tissues, energy deposition profiles obtained from the optical model for irradiation at 532 nm are converted into predictions of the temperature rise induced by the KTP laser, as a function of subsurface depth, Δ*T*(*z*, *t* = 0). According to theory, the corresponding radiometric transient Δ*S*(*t*) can be expressed as

(2) $$\Delta S\left(t\right)=\;\int_{z=0}^{\infty }K\left(z,t\right)\;\Delta T\left(z,t=0\right)\;dz.$$

Here, the kernel function *K*(*z*, *t*) accounts for both heat diffusion dynamics and spectrally dependent attenuation of the subsurface IR emission by the superficial tissue layers [16].

### 2.6. Assessment of Skin Properties by Inverse Monte Carlo Technique (IMC)

Skin structure and composition at the selected test sites is assessed by matching the experimental DRS spectra and PPTR signals with the corresponding predictions of our numerical model. The solution is sought by iterative multi-dimensional minimization of the residual norm using the nonlinear least-squares algorithm (function “lsqnonlin” in MATLAB Optimization Toolbox, Mathworks). For simultaneous fitting of the DRS and PPTR data, we combine the respective residual norms into a single cost function with a merging factor *M* (i.e., ε = ε_DRS_ + *M ε*_PPTR_). In the presented examples we use *M* = 10, which was found suitable for the problem at hand [14].

As mentioned above, the intact skin site is analyzed first by optimizing 14 free parameters of the corresponding optical model (Section 2.3): The epidermal and dermal thicknesses (*d*_epi_, *d*_pap_, and *d*_ret_), melanin content (*m*), papillary and reticular blood contents (*b*_pap_ and *b*_ret_) and their oxygenation levels (*S*_pap_ and *S*_ret_), *β*-carotene concentration (*c_β_*), scattering parameters in epidermis and dermis (*a*_epi_, *p*_epi_, *a*_der_, and *p*_der_) and subcutis scattering amplitude (*A*). In the subsequent analysis of the bruised site, most of these parameters are fixed to the values assessed from intact skin, while those that are expected to vary significantly upon bruising are assessed independently for each time point (Section 2.4): Two dermal blood contents (*b*_pap_ and *b*_ret_) and their respective oxygenation levels (*S*_pap_ and *S*_ret_), bilirubin concentration (*c*_bili_), and dermal scattering amplitude (*a*_der_).

**Figure 2:**
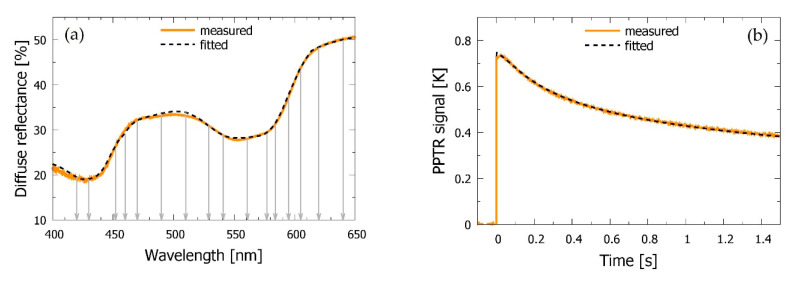
(**a**) Diffuse reflectance spectroscopy (DRS) spectrum and (**b**) pulsed photothermal radiometry (PPTR) signal as obtained from the intact skin site near the bruise in subject *a* (solid orange curves) and the best-fitting model predictions (dashed lines). Vertical gray arrows in (**a**) indicate the wavelengths included in the Inverse Monte Carlo (IMC) analysis of the DRS data.

In either case, only the initial 1.5 s of the transient PPTR signal and diffuse reflection values at 16 strategically selected wavelengths between 420 and 640 nm are used in the optimization process (see Figure 2a) in order to control the computational load of the numerous MC runs involved in this high-dimensional optimization process. In this way, one IMC run for assessment of 14 skin parameters from experimental data takes 10–12 min on a graphical node of a computational cluster with 16 CPU cores, 128 GB of RAM, and 2 high-performance graphics cards (Nvidia GeForce GTX TITAN X). Meanwhile, the analyses of bruised sites, with 6 free parameters, take around 5–7 min. However, because of the stochastic nature of the IMC procedure, each analysis must be repeated 5–12 times with different (randomized) initial parameter values.

## 3. Results

### 3.1. Intact Skin

Figure 2 presents one DRS spectrum and PPTR signal as obtained from the intact skin site in subject *a.* In agreement with our earlier analyses using a very similar approach [19], the best-fitting predictions of our four-layer optical model (dashed lines) match the experimental data very well. This is confirmed by regression analysis, yielding values of *R*^2^ = 0.998 for both DRS and PPTR data.

**Figure 3:**
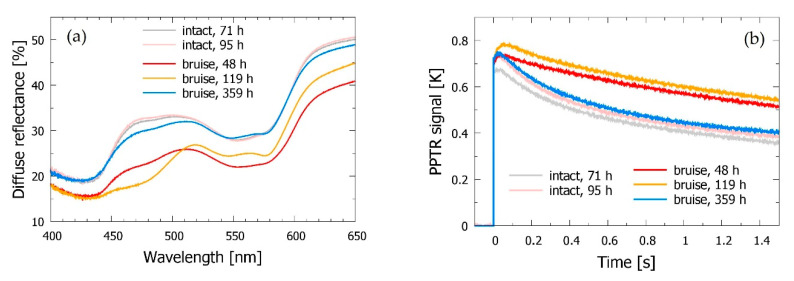
(**a**) DRS spectra and (**b**) PPTR signals obtained from the intact (gray and pink lines) vs. bruised skin site in subject *a* at different times after the injury (see the legends).

However, we can occasionally observe some day-to-day variations of the measured DRS and PPTR signals, most likely originating from uncontrolled changes in skin blood circulation and oxygenation levels. One example of such variations can be seen in Figure 3 (gray and pink lines). From the point of view of robustness of the subsequent analysis, we have therefore found it very beneficial to obtain the baseline skin parameter values from a simultaneous fit of 2–3 measurements performed on the same intact site on different days [23].

The results of such combined analyses for our three subjects are presented in Table 2. All assessed values lie within the anatomically and physiologically plausible ranges for human skin, in line with our earlier experience with similar analyses [19,21,22,23]; e.g., the total thickness of skin in subject *a* amounts to 1.0 mm, which falls perfectly into the range of 1.1 ± 0.2 mm as reported for the front side of the thigh in young women (age 25–29 years) [37]. Next, the low melanin volume fractions, *m* = 0.4–0.5%, are consistent with the fair skin type and minimal sun tan observed in all three subjects. Note that the measurements were performed in March and April when the melanin content in our geographical area is at its seasonal minimum [15,19], especially at the included test sites which were covered with clothes through most of the winter.

Further on, the blood volume fractions in the papillary dermis (*b*_pap_) are consistently higher than in the underlying reticular layer (*b*_ret_), while the opposite is true for the corresponding oxygenation levels, in agreement with presence of the superficial capillary network [19]. Specifically, the results indicate that the cutaneous blood status in subject *a* did not change substantially between the selected time points. This is consistent with the small differences seen in the corresponding DRS spectra (Figure 3a), but was not necessarily the case for other subjects and measurement sessions [23].

### 3.2. Bruised Skin

Both DRS spectra and PPTR signals obtained from the bruised site display large differences with respect to the nearby intact site, as well as prominent day-to-day variations (see Figure 3).

**Figure 4:**
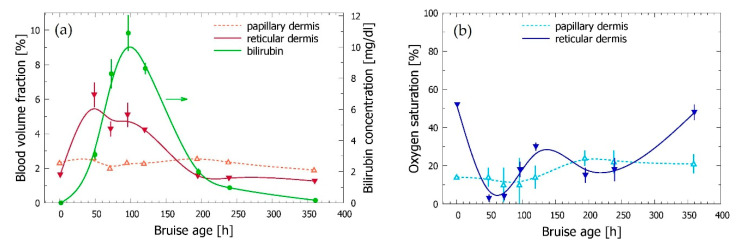
Time evolutions of the bruise model parameters as assessed from analyses of the data obtained from subject *a* at different times after injury: (**a**) Volume fractions of blood in the papillary and reticular dermis, and concentration of bilirubin when confined to the dermal layers; and (**b**) oxygenation levels of blood in both dermal layers. (The curves have no theoretical basis and serve only as a guide to the eye).

The general trends observed in the DRS spectra of bruised skin (Figure 3a) match those reported in previous studies from other groups [7,8,38]; e.g., a significant decrease of diffuse reflectance can be seen across the presented spectral range, due primarily to the higher dermal blood content. This is supported by the main features of the corresponding PPTR signals (Figure 4b). Namely, the larger temperature rise and more gradual relaxation toward the baseline temperature are consistent with enhanced absorption of the green laser light, especially in deeper skin layers [16,18].

In addition, the DRS data indicate strongly enhanced absorption of blue/green light (λ = 450–520 nm) 3–4 days after the injury (see yellow line, *t* = 119 h), which indicates presence of an additional absorber, such as bilirubin.

It is also worth noting that 15 days after the injury, when the bruise has almost completely resolved, both DRS spectra and PPTR signals return towards the respective data obtained from the intact skin site (blue lines, *t* = 359 h). However, some excess absorption can still be noticed in the blue/green spectral band and in the PPTR data (Figure 3a,b, respectively). This is consistent with the residual discoloration visible in Figure 1 and with an earlier independent report [39].

### 3.3. Time Evolution of the Bruise

After establishing the general properties of the investigated skin site (see Table 2), we can proceed to analysis of the bruised site. By performing independent IMC analyses of the data acquired at different time points, we can obtain an insight into time evolution of the free parameters of the corresponding optical model (Section 2.4).

As can be seen in Figure 4a, the results show a substantial increase of the blood content in reticular dermis (solid red triangles) compared to the value obtained for the intact site (plotted at *t* = 0) in the first two days after the injury. This is a clear indication of the hemoglobin diffusing in large amounts from the hemorrhage site into lower dermis. This interpretation is further supported by the dramatic drop of the reticular blood oxygenation (Figure 4b, blue), from the usual level of 50–55% in intact skin [19] to only a few % two days after the injury. One to two weeks later, both variables are gradually returning towards their initial values, characteristic for healthy skin. Meanwhile, the blood content and oxygenation level in the papillary dermis (open red and cyan triangles in Figure 4a,b, respectively) show significantly smaller changes over the course of the bruise healing process.

Of most interest is, however, the behavior of the bilirubin concentration (solid green circles), which rises steadily to its peak value on day 4. The indicated lag behind the reticular blood is plausible, because bilirubin is formed only by biochemical degradation of the extravasated hemoglobin. A more detailed discussion of the observed trends and values can be found in Section 3.4 and Section 4.

In the optical model used for the above analysis, bilirubin was confined to the dermal layers, same as seen in earlier similar studies [7,8,11]. This assumption was based on the fact that bilirubin is formed from hemoglobin which, due primarily to the size of its molecule, cannot diffuse across the epidermal-dermal junction (EDJ) [40].

**Figure 5:**
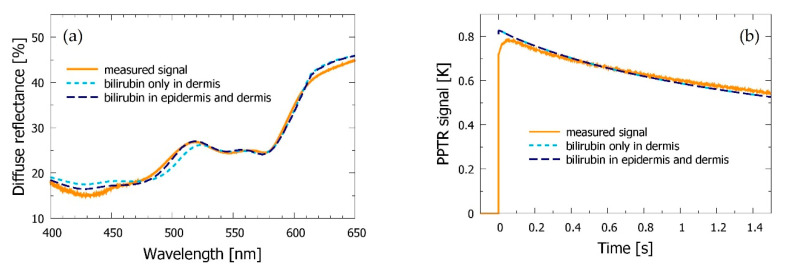
(**a**) DRS and (**b**) PPTR signals acquired form the bruise in subject *a* at 119 h after injury (orange lines). Dashed lines present the best-fitting model predictions when bilirubin is allowed only in the dermis (light blue), and in both epidermis and dermis (dark blue).

However, we have noticed that this approach often provided a poor match with experimental DRS spectra, especially in test sites with relatively thick epidermis (see Figure 5a). Therefore, we have also explored the opposite option, allowing for presence of bilirubin in the epidermal layer. Such a modification enabled an improved match with the measured DRS spectra in many cases, especially in the range of bilirubin absorption (i.e., 475–525 nm); e.g., in the example presented in Figure 5a the described change led to an increase of the correlation coefficient from *R*^2^ = 0.979 to 0.992. Meanwhile, the match obtained for the PPTR signals was not affected (Figure 5b). This can be attributed to the fact that the 532 nm laser light used in our PPTR measurements is only weakly absorbed by the bilirubin.

**Figure 6:**
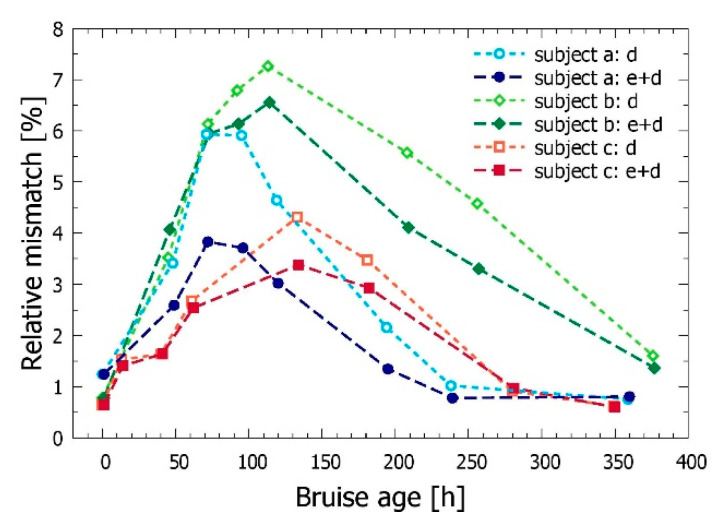
Relative mismatch (δ, Equation (3)) between the measured and model-predicted diffuse reflectance values in the IMC analyses of the bruises in all three subjects at different times after the injury. Open symbols mark the results of the model with bilirubin only in the dermis, full symbols correspond to the bilirubin present in both epidermis and dermis.

Moreover, we have found that the improved match in the DRS spectra obtained by allowing for bilirubin presence in the epidermis was substantial and robust. This is evidenced in Figure 6 as the consequent reduction of the relative mismatch in the analyses of bruises for all included subjects at times between 2 and 10 days after the injury, when the bruises were most developed. The relative mismatch was computed as the average of the absolute values of the differences between the measured and model-predicted signal values, divided by the former:

(3) $$\delta =\frac{1}{N}\sum_{i=1}^{N}\frac{\left|x_{i}-x_{i}^{\prime }\right|}{x_{i}}.$$

### 3.4. Bilirubin Allowed in Both Epidermis and Dermis

Based on the arguments presented in Section 3.3, we apply in the following analyses the optical model which includes bilirubin in the epidermis and both dermal layers. This decision is further supported by a recent report that dye molecules of similar molecular weight and partition coefficient to bilirubin can penetrate through the EDJ [41]. In addition, bilirubin was detected in the epidermis of rat, mouse, and guinea pig skin [42].

**Figure 7:**
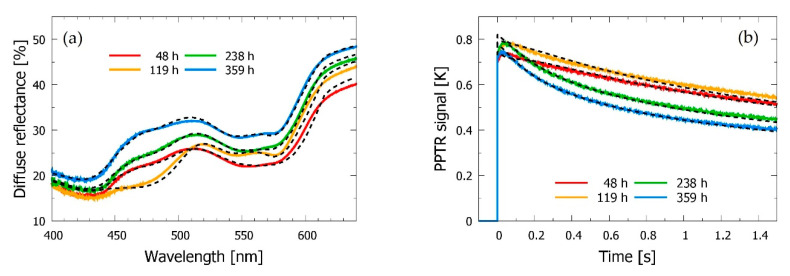
(**a**) DRS spectra and (**b**) PPTR signals obtained from a bruised site in subject *a* at four different time points (see the legend), and the corresponding model predictions (dashed lines).

As illustrated by Figure 7, this model follows the variations in both DRS and PPTR data during the bruise healing process very well. The relative mismatch between the experimental and model DRS does not exceed 4% (see Figure 6) and the R^2^ values are between 0.981 and 0.998 for the DRS part and 0.952–0.995 for the PPTR.

**Figure 8:**
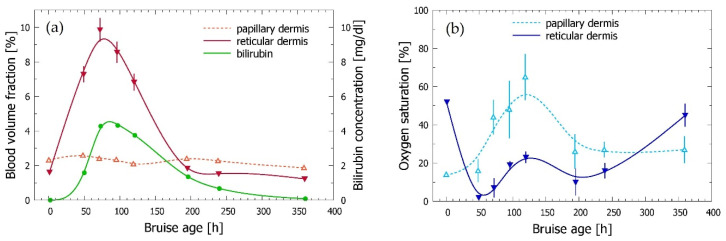

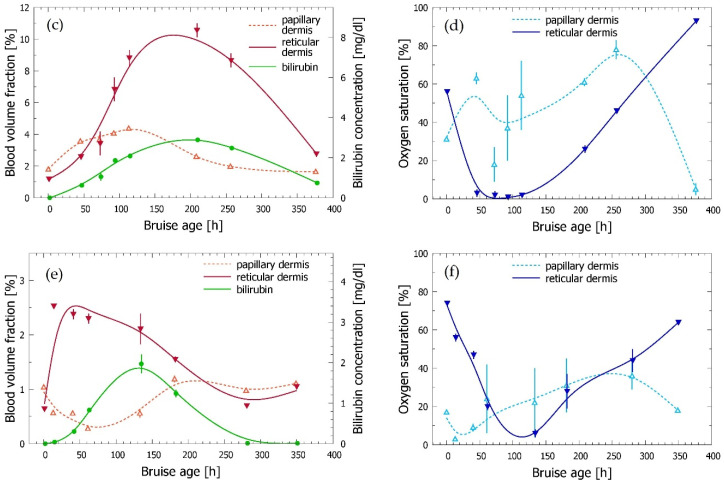
Temporal evolution of the bruise model parameters with bilirubin present in both epidermis and dermis as assessed from measurements in subjects *a* panels (**a**,**b**), *b* (**c**,**d**), and *c* (**e**,**f**). The curves serve only as a guide to the eye. The error bars represent standard deviations from 6 IMC runs.

As can be seen in Figure 8a,b, the described modification of the optical model does not affect the main trends observed in our initial analysis of the same experimental data (Figure 4); i.e., the substantial increase of the reticular blood content compared to the value in intact skin, accompanied by a dramatic drop of the reticular oxygenation level in the first days after the injury (solid red and blue triangles, respectively), and the somewhat delayed appearance of bilirubin (green circles). Similarly, the papillary blood content remains relatively unaffected by the bruise onset and subsequent healing process (open red triangles).

On the other hand, the described modification of the optical model led to certain quantitative changes with respect to the provisional results in Figure 4. The most notable are the reduced bilirubin concentration values, which now peak just above 4 mg/L (compared to the former ~11 mg/L). This is logical, since any chromophore will have a stronger influence on the DRS spectra when located closer to the skin surface. Interestingly, however, these results also exhibit significantly smaller standard deviations (as assessed from several IMC runs). This could be an additional indication that the modified optical model is more realistic, thus leading to better convergence of the IMC optimization procedure.

An opposite effect is observed for the reticular blood content (solid red triangles), which now peaks at ~50% higher values. This change is not as plausible as the one discussed just above, but might result from the overlapping absorption spectra of the hemoglobin and bilirubin in the blue/green spectral range, combined with screening of the incident light by the epidermal bilirubin.

The oxygen saturation in reticular dermis (solid blue triangles) behaves remarkably similar in both results. In our opinion, this demonstrates the robustness of the underlying differential measurement approach (as this parameter is defined as the ratio of two concentrations). On the other hand, we are not quite sure why the same argument does not apply also to the oxygen saturation in the papillary layer (open light blue triangles), which on average assumes higher values than in the initial analysis.

In the rest of Figure 8 (panels c–f) we present the results obtained by applying the same approach to experimental data from subjects *b* and *c*. This serves as an additional test of robustness of our approach, while at the same time providing a provisional insight into individual variability of the dynamical processes in self-healing bruises.

The obtained values clearly indicate rather smooth transient changes for most presented parameters, in many cases substantial compared to the values found in the intact site. This is followed by gradual relaxation towards the respective initial values. In all three examples, the relative changes of the blood content in reticular dermis (solid red triangles in Figure 8a,c,e) are significantly larger than those in the papillary layer (open triangles). A similar relationship can be seen also between the respective oxygenation levels (see panels b, d, and f). Specifically, the double-through (w-shaped) behavior of the reticular blood oxygenation indicated for subject *a* (Figure 8b) has been reproduced despite the introduced modification of the model structure. We therefore believe that it is most likely real, indicating a secondary hemorrhage from the damaged blood vessel.

Meanwhile, bilirubin concentration (solid green circles) increases smoothly in all three examples, from the initial value of 0 to its peak several days later, followed by a monotonic decrease. Complete removal of the bilirubin can be seen in the last data points for subjects *a* and *c*, but not in the case of subject *b*, where the bruise appears to develop and heal at a much slower rate (panel c).

**Figure 9:**
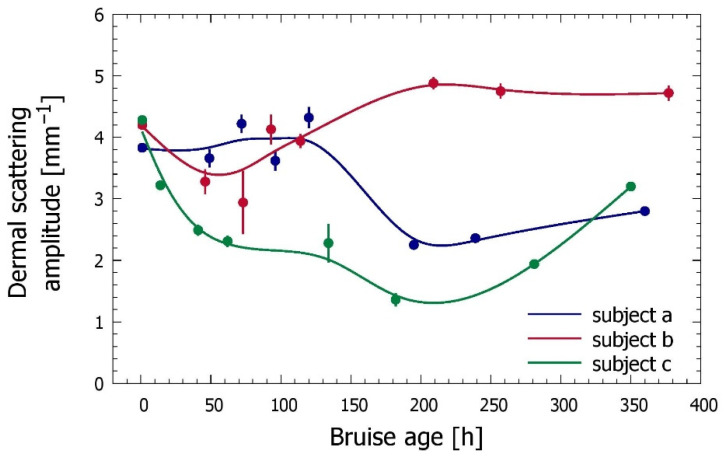
Comparison of the assessed time evolution of the dermal scattering coefficient (*a*_der_) in three included subjects.

Finally, Figure 9 presents the assessed time evolutions of the dermal scattering amplitude, *a*_der_. Significant deviations from the values obtained for the intact site (*t* = 0) can be seen during the bruise onset and healing. All three timelines indicate a significant reduction of dermal scattering at some point during the bruise evolution, but they exhibit important individual variations. More detailed analyses of the obtained bruise model parameter values and time evolutions are presented in Section 4.

## 4. Discussion

In the present study, we use an augmented version of the approach for noninvasive assessment of skin structure and composition, involving DRS and PPTR measurements coupled with numerical modeling of light transport in a four-layer skin model, developed recently in our group [19]. The modifications necessary for its application to monitoring of dynamical processes in traumatic bruises include explicit accounting for the presence of *β*-carotene in the intact and bilirubin in bruised skin. In addition, the thickness of the papillary dermal layer is adjusted individually, rather than being fixed to an anatomically plausible value, for greater flexibility of the skin model. Next, in order to achieve sufficient robustness of the involved inverse analysis, measurements from an intact skin site in the vicinity of the bruise are characterized first by the four-layer model of healthy skin. Only selected parameters of the optical model are then allowed to vary to match the data obtained from the bruised site, and finally, in doing so, we combine several measurements from the intact site, acquired on different days.

For most bruise model parameters, the obtained values indicate gradual transient changes, followed by gradual relaxation towards their initial values (Figure 8 and Figure 9). Given that each time point was analyzed separately from the others, using experimental data acquired on different days, the relatively small amount of scatter around the smooth timelines is a strong indication of the robustness of our approach. The larger standard deviations seen for oxygen saturation in the papillary dermis (*S*_pap_) can be attributed primarily to the small thickness of this layer compared to the underlying reticular dermis, confounded by the fact that oxygenation of the latter (*S*_ret_) is assessed from the same spectral features.

The observed trends and values are also in very good agreement with the general understanding of the underlying processes and independent reports from other groups. Of most relevance for this study, the blood content in the reticular dermis (*b*_ret_) increases substantially over the first days after the injury, followed by a monotonic decrease towards the initial values as the bruises heal (Figure 8a,c,e). This is certainly an expected result, as the subsurface hemorrhage releases hemoglobin into lower dermis, from where it diffuses through the dermal matrix, while being also biochemically decomposed and removed through the lymphatic system.

The achieved peak values range from 2.5% in subject *c* to ~10% in the other subjects, which is 4–9 times higher than the corresponding values in the nearby intact site (0.6–1.7%, see Table 2). Large individual variations can also be seen between the time intervals required to reach the peak values. These amount to 1–2 and 2–3 days in subjects *a* and *c*, respectively, but extend to 6–8 days in subject *b*. A similar variance of individual timelines, while universally showing a gradual decrease of the excess hemoglobin content as the bruise resolves, was reported by Duckworth et al. [43] and Kim et al. [8]. Such a variability could result from many uncontrolled factors, from the severity of the impact force, size, and structure of the injured blood vessel and the surrounding tissue, to the dynamics of RBC lysis, hemoglobin diffusion, and even dynamics of its decomposition and removal.

Kim et al. [8] also used numerical modeling of light transport in a multi-layer model of skin and found a good match with experimental data at blood volume fractions up to 60% (typically 2 days after injury). The values reached in our present study are significantly lower in comparison. However, in our analyses of the most severe bruises (not included in this report), we have also obtained values above 40%.

In comparison with *b*_ret_ (see above), blood volume fraction in the papillary dermis (*b*_pap_) exhibits much smaller changes relative to the corresponding initial values throughout the bruise evolution. In all three presented examples, *b*_ret_, which is initially smaller than *b*_pap_, thus, exceeds the latter by a considerable margin in the phase of most developed bruise. This is in perfect agreement with the hemoglobin diffusing gradually from the relatively deep hemorrhage site towards the skin surface while at the same time being decomposed and removed, which maintains a vertical gradient of its local fractional content.

At later times, when the excess hemoglobin has been largely removed, the value of *b*_ret_ invariably drops again to (or below) the current values of *b*_pap_. In subjects *a* and *c* this occurs 8–10 days after the injury, while in subject *b* this has not yet occurred at the last documented time point (~16 days after injury). However, the indicated trend strongly suggests that this would happen in another day or two.

Under closer scrutiny, the three presented timelines *b*_pap_(t) exhibit some individual differences. It should be noted that the papillary dermal layer is known for large variations of blood perfusion due to uncontrolled physiological processes, such as thermoregulation [19,23,44,45]. On the other hand, additional processes directly related to bruising, such as inflammation, may also contribute to the observed trends [46].

The oxygen saturation level in reticular dermis (*S*_ret_) displays a dramatic reduction over the first 2–5 days after the injury, after which it gradually returns to the values typical for intact skin (Figure 8b,d,f). Starting from the values of *S*_ret_ = 53–75%, characteristic for intact skin [19,23], the relative changes to the lowest values of 2–5% are even larger than those seen for *b*_ret_. This is in perfect agreement with depletion of oxygen from the hemoglobin released from the extravasated and subsequently lysed RBC. This effect fades away when the amount of excess hemoglobin is reduced to the degree where the vascular blood again begins to dominate the assessed oxygen saturation value.

Similar reductions of the dermal oxygen saturation levels in bruised skin were reported earlier; e.g., Randeberg et al. [7] reported a decrease of extra-vascular oxygen saturation by 10–30% when compared to intra-vascular saturation in the upper dermis in one third of the analyzed bruises, with the lowest assessed value of ~20%. More recently, Kim et al. [8] found a decrease from 60% to 40% in three days after injury by including extravascular blood in the model.

Because both groups reported considerably smaller relative changes than those seen in all three examples of present study, we speculate that our approach may be more successful in differentiating between the two dermal layers, perhaps owing to the high sensitivity of the PPTR measurements for depth distribution of selected absorbers. In our results, oxygen saturation in the papillary dermis (*S*_ret_), namely, does not drop in parallel with *S*_ret_, but rather indicates (on average) a weak transient increase. This could be tentatively interpreted as a sign of inflammation, but on the other hand, the papillary dermal layer is prone to uncontrolled variations [19,23], and its small thickness may adversely affect the accuracy of its characterization as already discussed above. Nevertheless, the larger values and smaller relative changes reported in refs. [7,8] suggest that those results might represent weighted averages of the values from the upper and lower dermal layers.

The bilirubin concentration increases smoothly from the initial value of 0 in intact skin to the maximum concentration reached after 3–4 days in subject *a*, 5–6 days in subject *c*, and 8–9 days in subject *b*. Most importantly, its peak always occurs after the observed blood content peak (by approximately 1–4 days). This is correct, because bilirubin is formed only by decomposition of extravasated hemoglobin (via an intermediary substance, biliverdin).

Our results correspond very well to literature data, which stated that the bilirubin content (or Δb* value in the case of colorimetric studies) typically peaks at 3–5 days [5,8,9,38,47]. However, longer times required to reach the bilirubin peak were also reported, i.e., 4–8 days [1] and up to 10 days [43], which supports the significantly slower dynamics seen in our subject *b*.

Furthermore, all our examples demonstrate a subsequent gradual removal of the bilirubin. In subjects *a* and *c*, bilirubin cannot be detected 12–14 days after the injury, but it is evidently still present after 16 days in the case of subject *b*. This is an independent demonstration that the latter bruise has not completely resolved during the measurement interval, thus supporting the indication of slow dynamics of *b*_ret_ in this subject (Figure 8c).

The rate at which bilirubin is removed from bruised skin appears to vary between our three subjects. Such variations may reveal individual differences in the immune system response, but could also arise from the complex interplay with the dynamics of hemoglobin removal and spatial diffusion of both species.

The bilirubin concentration values in our results also correlate well with literature data. In a recent study using spectrophotometry (also known as bilirubinometry), Abdy et al. [48] reported mean bilirubin concentrations of 3.8 mg/dL. Randeberg et al. [11] modeled the bilirubin contribution at 1%, which amounts to 5.4 mg/dL, while Kim et al. [8] found a good match with measurements at bilirubin concentrations between 5 and 40 mg/dL. However, unlike the present report, the latter study included cases of severe bruising. Our preliminary analyses of such cases resulted in values around 20 mg/dL (unpublished).

In addition to oxy- and deoxyhemoglobin, our current model accounts only for their most common decomposition product, bilirubin. For improved versatility of the analyses, one might want to include other chromophores linked to bruise evolution, such as hemosiderin or methemoglobin, which can be responsible for brown discoloration in some bruises [7]. However, this would require inclusion of longer wavelengths in the DRS measurements and model analysis.

The values of dermal scattering amplitude obtained from intact skin sites (see Table 2) are very similar between our three subjects, and in perfect agreement with the range of values from literature (*a*_der_ = 4.6 ± 1.4 mm^−1^) [15], as well as those obtained using a very similar approach in other human volunteers of similar age [19].

Significant deviations from the baseline values are clearly indicated during the bruise onset and healing (Figure 9). While a significant reduction of dermal scattering is indicated in all three subjects at some point during the bruise evolution, there are also important differences between the three results.

The largest and most persistent decrease of *a*_der_ is indicated for the bruise in subject *c*. Compared to the initial (intact) value it drops by nearly 70% by day 7 after injury, and returns only gradually when the bruise is almost completely healed. We believe that the indicated decrease of dermal scattering is real and can be linked directly to the presence of extravasated hemoglobin in the interstitial fluid. This reduces the refractive index mismatch with the collagen matrix [49], in the same way as application of optical clearing agents [50,51]. The subsequent conversion of the hemoglobin to bilirubin most likely does not make a big difference in this regard, which may help explain why this effect persists longer than the presence of excess hemoglobin seen in Figure 8a.

The different timelines indicated in Figure 9 for subjects *a* (where the scattering amplitude hovers around the value for intact skin but drops by ~50% 7–8 days after injury) and *b* (where it exhibits an initial drop by 20–30% followed by an increase to 25% above to the initial value five days later) most likely indicate a combined effect of the “index matching” effect described just above and another one which enhances dermal scattering.

One candidate for the latter could be the extravasated RBC, which require certain time before becoming completely hemolyzed, especially when spilled at a high rate [46,49]. The bruised site may also experience increased water content in the interstitial fluid (edema), which would increase the refractive index mismatch with the collagen matrix, and thus enhance the scattering. In addition, bilirubin was shown to increase the aggregation rate of collagen and accelerate the lateral growth of collagen fibrils in vitro [52]. Finally, the impact force inducing the hemorrhage may also directly affect the collagen structure, which is remodeled as the bruise heals [53]. Further research would be required to determine which effects dominate in general and in any specific example. In principle, however, their different temporal profiles could lead to very diverse dynamics of their combined effect on dermal scattering.

For assessment of the properties of intact skin, which serve as a baseline for subsequent assessment of the bruise dynamics, different approaches can be conceived in addition to the one applied in the present report (Section 3.1). In a short dedicated study, we have tested three such approaches, which involve analysis of the DRS and PPTR data from measurements performed in (a) a nearby intact healthy site on 2–3 different days (i.e., the approach used in this report); (b) the bruised site after the bruise has completely resolved; and (c) a combination of the two [23]. The first approach may successfully account for the daily variations of the dermal blood contents and oxygenation levels, and thus greatly improve the robustness compared to the analysis of a single measurement. However, a legitimate doubt exists regarding potential differences in the skin structure and composition between the selected intact site and the actual bruise.

The later argument evidently favors delayed measurements at the actual bruised site. However, since we see that even gross bruise dynamics involving hemoglobin and bilirubin can sometimes take longer than the anticipated two weeks (Figure 8c), adhering to approaches (b) and (c) would require keeping all volunteers in the protocol ~2 weeks longer. Moreover, such protocols would be even more impractical in the target forensic applications. They would namely require that the subject (e.g., a victim of a violent crime) attends two measurement sessions separated by 3–4 weeks, and the data analysis could only begin after the second one. Luckily, however, our comparison study did not show any significant advantages of such longer and more complicated protocols regarding the quality of subsequent results [23].

One practical limitation to note is, however, the large computational effort required for the characterization of the intact skin site by simultaneous analysis of 2–3 measurements. This, namely, greatly increases the dimensionality of the involved IMC optimization process, which extends the computation time to ~80 min for a single IMC run using our current hardware as described above (Section 2.6). We anticipate, however, that this process might be expedited in the future by exploring machine learning technology, e.g., artificial neural networks [54] or random forests approach [55].

Finally, Figure 6 shows that the relative mismatch (Equation (3)) between the measured and simulated DRS achieved in our IMC analyses is largest for the bruise ages of 2–8 days. This could be attributed to the fact that the depth distribution of the extravasated hemoglobin at those times deviates the most from the step-wise constant profile, dictated by our four-layer optical model with optically homogeneous layers. Moreover, the depth profile of the bilirubin concentration, which most likely resembles that of hemoglobin, is assumed to be constant throughout the skin. We are therefore exploring the use of mathematical models of hemoglobin diffusion and conversion to bilirubin, similar to the one proposed by Randeberg et al. [7]. This enables analytical expression of the hemoglobin and bilirubin profiles at different times, which can be easily accounted for in the MC modeling [34].

## 5. Conclusions

The presented methodology, combining DRS and PPTR measurements with IMC analysis involving a four-layer model of human skin, enables noninvasive characterization of most interesting chromophores in traumatic bruises and objective monitoring of their temporal evolution. The results of our test runs performed on three human volunteers over 16 days clearly indicate a gradual, yet considerable increase of the dermal blood content and reduction of its oxygenation level in the reticular dermis in first days after the injury. This is followed by the delayed emergence of bilirubin and subsequent relaxation of all variables towards the initial values characteristic for healthy skin, with the characteristic times and values matching those from literature. The presented approach, thus, offers another viable option for studies of the bruise healing dynamics, which appear essential for future development of a technique for objective assessment of the bruise age.

## Figures and Tables

**Table 1:** Basic information on the included subjects and bruises.

Code	Subject: Gender	Age	Anatomic Location	Cause of Trauma	Measurement Times
*a*	female	29 y	thigh, bellow the hip	hit into a table	48–359 h
*b*	male	30 y	outer side of the thigh	off-piste skiing	45–376 h
*c*	female	29 y	upper forearm	roller derby	13–349 h

**Table 2:** Characteristics of the intact skin site in all three subjects assessed by simultaneous analysis of the data obtained on two or three consecutive days. The dermal blood contents (*b*_pap_ and *b*_ret_) and oxygenation levels (*S*_pap_ and *S*_ret_) were allowed to vary, while the remaining parameter values were linked together (for the definitions please see Section 2.3). The indicated intervals are standard deviations from 5 IMC runs (omitted when smaller than 1 on the last decimal place).

	Thickness	Absorbers			Scattering
subject *a*					
epidermis	*d*_epi_ = 0.08 mm	*m* = 0.5%			*a*_epi_= 9.3 ± 0.2 mm^–1^
		*b*_epi_ = 0			*p*_epi_= 1.7
dermis	*d*_pap_ = 0.18 ± 0.01 mm	*b*_pap_ = 2.4%	2.3%		*a*_der_= 3.8 ± 0.1 mm^–1^*p*_der_= 1.6
		*S*_pap_ = 10 ± 1%	18 ± 2%		
	*d*_ret_ = 0.76 ± 0.02 mm	*b*_ret_ = 1.6%	1.7%		
		*S*_ret_ = 55 ± 1%	50 ± 1%		
		*c*_β_ = 1.7 ± 0.2 mg/L			
subcutis	–	–			*A* = 0.3
subject *b*					
epidermis	*d*_epi_= 0.10 mm	*m* = 0.4%			*a*_epi_= 7.7 ± 0.2 mm^–1^
		*b*_epi_ = 0			*p*_epi_= 1.7
dermis	*d*_pap_ = 0.14 ± 0.01 mm	*b*_pap_ = 1.9%	1.7%	1.8%	*a*_der_ = 4.2 ± 0.1 mm^–1^*p*_der_ = 1.5
		*S*_pap_= 38 ± 1%	34 ± 1%	22 ± 1%	
	*d*_ret_ = 1.02 ± 0.02 mm	*b*_ret_ = 1.2%	1.3%	1.0%	
		*S*_ret_= 52 ± 1%	42 ± 1%	76 ± 1%	
		*c*_β_ = 1.1 ± 0.2 mg/L			
subcutis	–	–			*A* = 0.4
subject *c*					
epidermis	*d*_epi_ = 0.07 mm	*m* = 0.4%			*a*_epi_= 9.7 ± 0.2 mm^–1^
		*b*_epi_ = 0			*p*_epi_ = 1.4
dermis	*d*_pap_ = 0.17 mm	*b*_pap_ = 1.0%	1.1%		*a*_der_= 4.3 ± 0.1 mm^–1^*p*_der_ = 1.9
		*S*_pap_ = 12 ± 1%	23 ± 1%		
	*d*_ret_ = 0.70 ± 0.02 mm	*b*_ret_ = 0.6%	0.7%		
		*S*_ret_ = 90 ± 1%	58 ± 1%		
		*c*_β_ = 1.0 ± 0.2 mg/L			
subcutis	–	–			*A* = 0.3

## Data Availability

Publicly available datasets were analyzed in this study. This data can be found at https://repo.ijs.si/bruises/sensors_2021.git.
